# Supplementary material for: Biophysics and population size constrains speciation in an evolutionary model of developmental system drift
Source: PLoS Comput Biol. 2019 Jul 23;15(7):e1007177. doi: 10.1371/journal.pcbi.1007177 (PMC6677325; doi:10.1371/journal.pcbi.1007177)
Supplement: S1 Text — (PDF) [file pcbi.1007177.s001.pdf]

Supporting Information

Biophysics and population size constrains speciation in an evolutionary model of developmental system drift

Bhavin S. Khatri[1*¤], Richard A. Goldstein[2],

1 The Francis Crick Institute, London, United Kingdom
2 Division of Infection & Immunity, University College London, London, United Kingdom

¤Current Address: Department of Life Sciences, Imperial College London, Silwood Park, Ascot, United Kingdom
* bhavin.khatri@physics.org

Emergent properties of genotype-phenotype map

The properties of this genotype-phenotype map have been previously explored [S1]. So here we summarise its pertinent findings. Under a fixed environment (fixed fitness) adaptation occurs in a manner which is insensitive to initial conditions and to the same ensemble of states[1]. As shown in Fig.S1, the evolutionary simulations find solutions to the patterning problem, where it is clear that for a small scaled population size ($2N\kappa_F = 1$) the solutions fixed at any one time are less constrained, while as the population size increases ($2N\kappa_F = 50$) the solutions are more constrained. This is reflected in the time series of the fitness as shown in Fig.S2a, where we see that for $2N\kappa_F = 1$ the fitness of solutions are low, with large fluctuations and very often near the inviability threshold F^*, whereas for $2N\kappa_F = 50$ the fitness is higher and far more constrained. We see this also in the time series of the phenotype α (Fig.S2b), which shows that it is small with large fluctuations for $2N\kappa_F = 1$ and is larger and more constrained for $2N\kappa_F = 50$, which reflects the fact that a larger value of α corresponds to a more steep gradient of the morphogen, which in turn allows sharper patterning.

As was found in [S1], there are a large number of genotypic states that give good patterning and they all belong to the same solution to the patterning problem; the morphogen binds cooperatively with the polymerase to promote transcription when the morphogen concentration is large and turn off transcription when the morphogen concentration is small. As demonstrated by the histograms of the binding energy phenotypes in Fig.S2c&d, this solution in general requires E_{MB} and $\tilde{E}_{RM}$ to be small (strong binding) and that E_{RP} isn't too small, so that a switch-like mechanism exists to allow the polymerase to be attracted to the promotor in the presence of a large concentration of morphogen. The exact state of $\boldsymbol{G}$ determines these binding energies and then α is determined by the requirement that the threshold morphogen concentration which switches on or off transcription of TF occurs at the mid-point of the embryo. It is clear from Fig.S2c&d that some of the energy phenotypes are less important than others, in particular, $E_{MP}, E_{RB}, \tilde{E}_{MM}$ & $\tilde{E}_{RR}$ all have broad peaked distributions, even for large populations sizes, consistent with the neutral distribution of the binding energies, given this Hamming model, following a binomial distribution peaked about approximately about $\ell\epsilon/2$, and so indicating that the fitness constraints

[1] In other words the probability distribution of all binding and glue energies together with α is the same independent of initial conditions. In fact, the genotype-phenotype map is shown to be ergodic[S1]

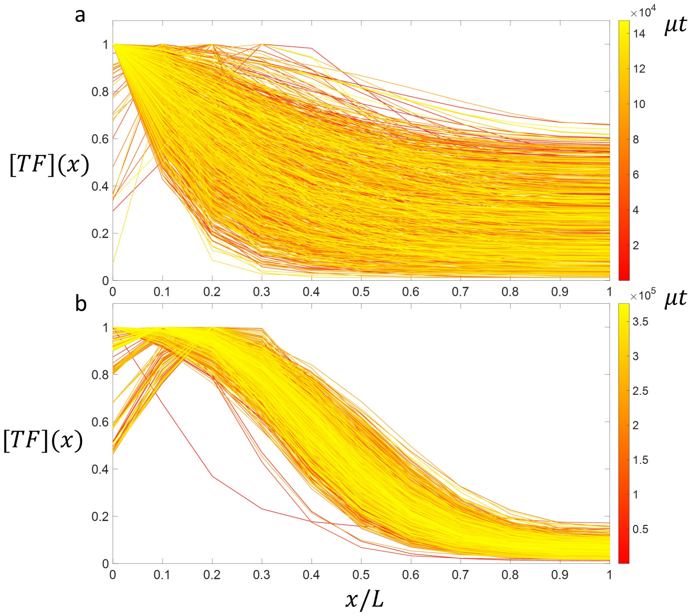

Fig S1. Typical spatial patterning profile of the output transcription factor $[TF](x)$ over the course of the evolutionary simulations, where early times are indicated by red, becoming progressively yellow at late times (as indicated by the colour bars); a) large population size $2N\kappa_F = 50$ and b) smaller population size $2N\kappa_F = 1$. the results show evolution gives solutions which are less well adapted.

on these phenotypes is relatively weak. Finally, an emergent property of the solutions is that there is a division of genotype space into a high sequence entropy region, where there are many sequences that code for slightly sub-optimal solutions corresponding to a small value of α, and a low sequence entropy, higher fitness region, which corresponds to a large value of α – this is what gives rise to the bistability visible in α for $2N\kappa_F = 50$ (see [S1] for more details). On long timescales, we see transitions between these regions, whilst on short timescales we mainly see exploration of each of these basins of attraction.

Different hybrid genotypes have different growth rates of DMIs

To examine in more detail the overall trends in the number of incompatibilities for each hybrid type, we plot the average number of DMIs as a function of divergence time μt in Fig.S3, where we have averaged over pairs of complementary genotypes (e.g. RmcA and rMCa), which have identical statistical properties. In Fig.S3, we first note that each hybrid-genotype behaves in a different, population size dependent manner. In general, there is an initial growth in the average number of DMIs as the divergence time increases, followed by a plateau or a slowing down of the growth. As denoted in the figure, we distinguish hybrid-genotypes based on the types of potential mismatch: R-type is characterised by a mismatch of the R locus with the M and C loci, M-type is a mismatch of M with the R and C loci (e.g., RmCa), C-type is a mismatch of C with R and M (e.g. rmCa) and α-type is a mismatch of the α locus with the rest of the loci. For each of the R-, M- and C-types, we assume the interaction with the α locus is relatively weak, so or example, the hybrid genotype Rmca or RmcA are both R-type.

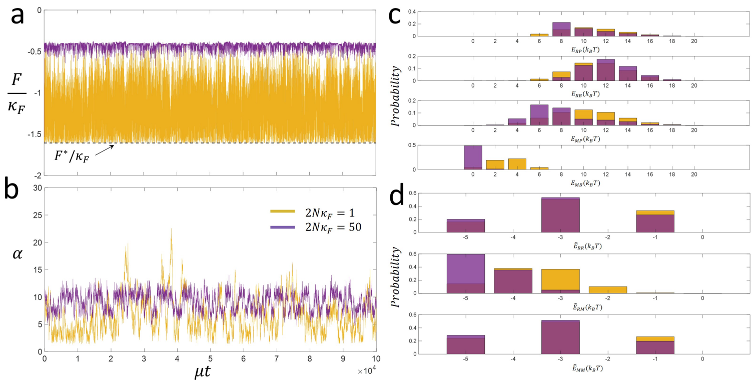

Fig S2. Equilibrium properties of phenotypes and fitness of genotype-phenotype map at the population sizes of $2N\kappa_F = 50$ (purple) and $2N\kappa_F = 1$ (yellow); a) Malthusian fitness (defined by Eqn.1&2 in the main text) over time, where we see whereas at large population sizes fitness is near the optimum, at low population sizes the fitness of solutions are sub-optimal and very commonly near the inviability boundary for functional vs non-functional spatial patterning; b) the morphogen steepness trait α where we see there is generally a broad variation at small population sizes, against more constrained variation at the larger population size; histogram of c) protein-DNA binding energy traits and d) of protein-protein binding energy traits, where we see that different binding energies are constrained to greater or lesser extent (indicating their selective or functional importance), which are further relaxed at smaller population sizes, indicating the increasing dominance of drift. In particular, as binding energies are determined by a sum of mismatches, the neutral distribution is a binomial distribution centred on $\epsilon_{pd}\ell_{pd}/2$ or $\epsilon_{pp}(\ell_{pp}+1)/2$, so that deviations from this distribution are a measure of the selective constraint.

In addition to the general trends pointed out in the main text, we see that the growth of DMIs is different for different hybrids; for small population sizes ($2N\kappa_F = 0.1$ & $2N\kappa_F = 1$), M-type, C-type and R-type dominate the growth of DMIs, in this order and with only a small difference between them, whilst α-type arise far more slowly; we might expect this since substitutions in α only tend to shift the pattern away from the mid-point of the embryo, which with the model of fitness defined in Eqn.7 (main text) only moderately affects fitness. As the population size increases, and at small times, we see that initially M-type DMIs arise more slowly relative to C-types and R-types, but at longer times there is a cross-over where M-type DMIs dominate R-type, which moves to longer times at increasing population sizes.

How can we understand this general behaviour? It is clear from Eqns. 7 & 8 (main text) that we need co-evolution of the relevant sequences to maintain these the important binding energies within certain constraints; e.g. the binding energy of R to the promoter P, E_{RP} mustn't be too strong and so on each line the sequences will co-evolve to maintain this constraint. From previous work on transcription factor DNA binding [S2, S3] we expect that the rate at which incompatibilities arise due a particular transcription factor binding site interaction j, is controlled by the product of the population size and the relative selection coefficient κ_j (which should be distinguished from the overall selection coefficient for the patterning phenotype κ_F); so the binding energy E_{MB} is under very strong selection to give high binding affinity of the morphogen to the 1st binding site, whilst for example, the binding energy E_{RP} is under weaker selection and so as $\kappa_{RP} > \kappa_{MB}$, we would expect incompatibilities due to this

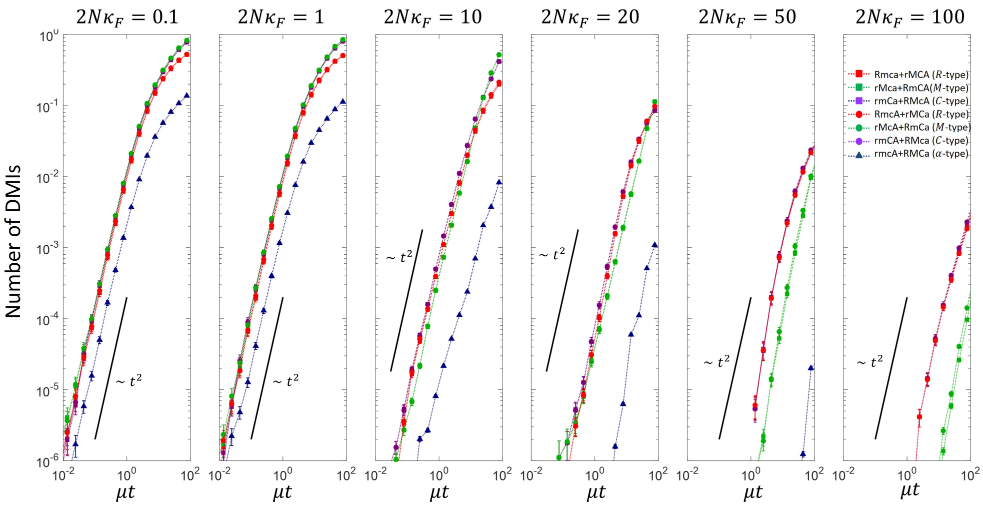

Fig S3. Plot of the number of DMIs for each hybrid genotype since divergence for different scaled population sizes. Complementary hybrid genotypes are summed over, since they have identical statistical properties.

interaction to arise more slowly when $2N\kappa_{MB} \gg 1$. Similarly, since $\kappa_{MB} > \kappa_j$, for all other interactions j, we would expect M-type hybrids to develop incompatibilities more slowly at scaled large population sizes, as observed in Fig.S3.

Null spectrum of DMIs

To test whether the parsimony decomposition of DMIs observed in Fig.5 of the main text, where 2-way DMIs are dominant, is not due to an inherent bias to detect 2-way DMIs of the method, we applied it to the case of randomly assigning incompatibilities to the different hybrid genotypes. This is easily accomplished by drawing random binary numbers from the Bernoulli distribution with a probability p that any hybrid is incompatible. For each random collection of incompatible hybrid genotypes the parsimony method is applied and the number of each type of n-way DMI is counted (as detailed in the Material and Methods of the main text); this is repeated 1000 times to calculate the average number of n-way DMIs shown below. To allow some comparison to the results of Fig. 5 of the main text, the value of p is varied according to the average fraction of incompatible hybrids found at different time-points from the simulations in the main text. Hence, the panels of Fig.S4 correspond to the number of n-way DMIs expected at different time-points for different scaled population sizes, where p is mapped into time using the sum of all curves in Fig.4 of the main text to calculate the fraction of incompatible hybrids at each time point. This null spectrum shows the opposite behaviour to that found in the simulations in the main text demonstrating that the results observed are not due to a random bias to find 2-way DMIs using the parsimony method.

Spectrum of DMI types

Spectrum of 2-way DMIs

In Fig.S5 we have plotted the number of 2-way DMIs of each type, where for example, I_{mc} is a 2-way DMI caused by an incompatibility between the M locus and C locus.

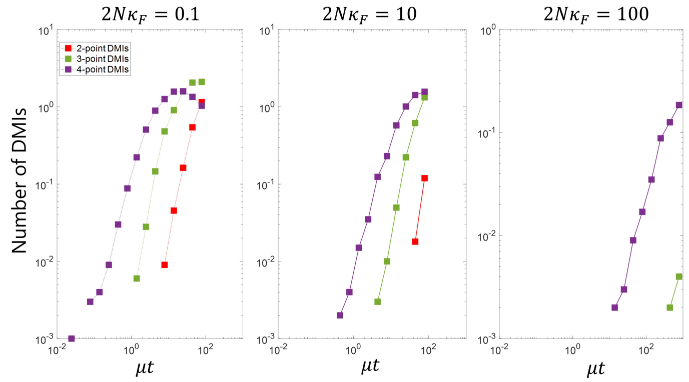

Fig S4. Parsimony decomposition of DMIs when incompatibilities are assigned randomly with a probability p that changes with time according to the fraction of hybrid genotypes that are incompatible from the simulations in main text.

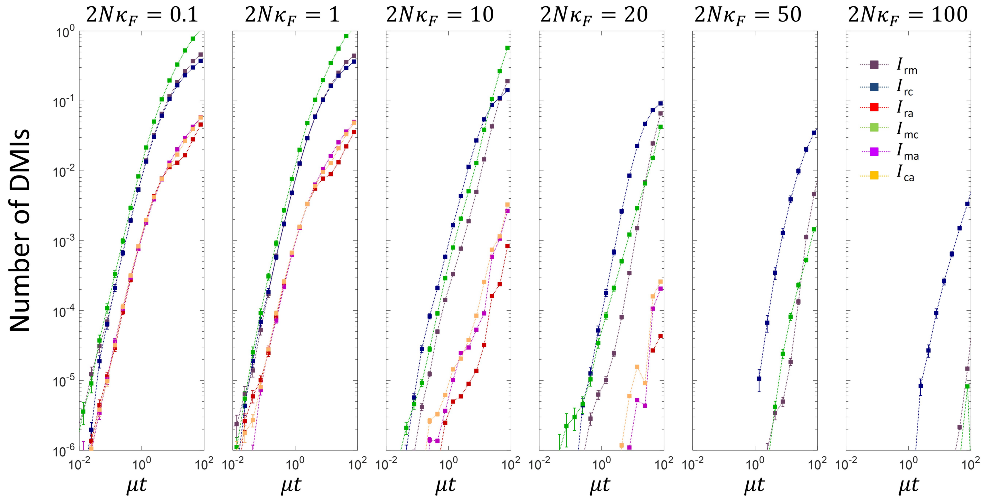

Fig S5. Plot of the spectrum 2-way DMIs vs divergence time for different scaled population sizes.

First, we note that at small population sizes the rate of increase of the different 2-way incompatibilities seem to cluster into two types; those that involve α and those that do not, where the latter arise more rapidly; this suggests that sequence entropy constraints are dominating for small populations, particularly at short times, though for the latter group there are some differences as discussed below. As the population size increases, we see that differences arise in the rate of growth between these different types of 2-way incompatibilities. Below we discuss these properties.

For each type of 2-way incompatibility there are 2 binding energy traits that could contribute. So increases in I_{mc} could be due to an incompatibility in the hybrid that can be traced to E_{MB} or E_{MP}; in this case, as the binding energy E_{MP} is almost neutral [S1], we would expect incompatibilities to arise predominantly from E_{MB}. Similarly, we expect I_{rc} to be dominated by E_{RP} and not E_{RB} and I_{rm} dominated by $\tilde{E}_{RM}$ and not $\tilde{E}_{MM}$ or $\tilde{E}_{RR}$, as E_{RP} is under strongest selection of the traits coded by the R & M loci. When it comes to incompatibilities involving the α-locus, there is no clear phenotypic trait that can be identified and as we will see the analysis of these

DMIs will not be so clear.

Examining Fig.S5, we see that as the scaled population size increases, the time for I_{mc} incompatibilities to arise sharply increases, while the time for I_{rm} increases less rapidly and I_{rc} even less rapidly. This corroborates a key prediction of a simple model of transcription factor DNA binding described in [S3], where traits under greatest selection should generate incompatibilities less rapidly, since the distribution of their trait values are further away from inviability in the common ancestor. As observed above, E_{MB}, which contributes most to I_{mc}, is under the greatest selection pressure and so as the population size changes these should change most rapidly. So we see that for large population sizes, it is not the phenotypic traits under the strongest selection that give rise to significant DMIs at short times, but those under a weaker selective constraint; traits under weaker selection will be affected more by the sequence entropic pressure for poorer binding affinities and so the common ancestor is more likely to be closer to the inviability boundary [2].

However, at small population sizes, $2N\kappa_F \leq 1$, all DMIs grow approximately quadratically at short times with a saturating form at long times, as also seen in Fig.5 of the main text. For small population sizes, the simple model of TF-DNA binding [S3] predicts that for the same sequence length and assuming the same threshold of inviability that the rate that incompatibilities arise should be approximately independent of selection and dominated by sequence entropy; if the sequence length of the trait increases, or the effective region of incompatibility is smaller, then we would expect incompatibilities to arise more quickly. As we see in Fig.S5, for small scaled population sizes and short times, the incompatibilities not involving α have similar behaviour except I_{mc}, which arrive more quickly; this is as expected as the effective mutation rate for I_{mc} to arise, will be double that for I_{rm}, since the binding of the morphogen to DNA involves 10 sites (nucleotides), vs 5 sites for protein-protein interaction. On the other hand I_{rc} also has 10 nucleotides, but arises more slowly. We suggest this could be due to a different effective region of incompatibility, which could confound simple expectations, it is not clear how a single inviability threshold F^* effectively maps to these pair-wise incompatibilities, complicating the picture further.

The 2-way incompatibilities involving the α locus are more difficult to interpret, since there is no clear trait in the patterning model associated with an interaction solely between the α locus and R, M, or C loci. As 2-way DMIs involving α are typically an order of magnitude smaller than the other DMIs they do not have a large impact on the number of DMIs.

Spectrum of 3-way & 4-way DMIs

In Fig.S6, we have plotted the 3-way DMIs as a function of divergence time μt, where the panels from left to right represent increasing scaled population size. We see that for small population sizes, 3-way DMIs between the R, M and C loci dominate at all times and in particular that the different types of DMIs of this type are all roughly equal, $I_{\mathsf{Rmc}} \approx I_{\mathsf{rMc}} \approx I_{\mathsf{rmC}}$. In addition, we see that all other DMIs arise more slowly and each of the 9 other types of 3-way DMIs are all again approximately equal. However, at larger population sizes this degeneracy is lifted amongst the different types of DMIs and different 3-way DMIs grow at different rates. How can we understand this general behaviour?

The patterning solution found in these evolutionary simulations involves the morphogen binding strongly to the first binding site recruiting the polymerase to bind

[2]However, if a trait is effectively neutral, i.e. that selection is sufficiently weak that for no trait values can incompatibilities arise, then these will not give rise to incompatibilities; the energy traits E_{RB}, E_{MP}, $\tilde{E}_{RR}$ and $\tilde{E}_{MM}$ have this property, as is evident by examining their marginal distribution functions which follow the neutral expectation [S1].

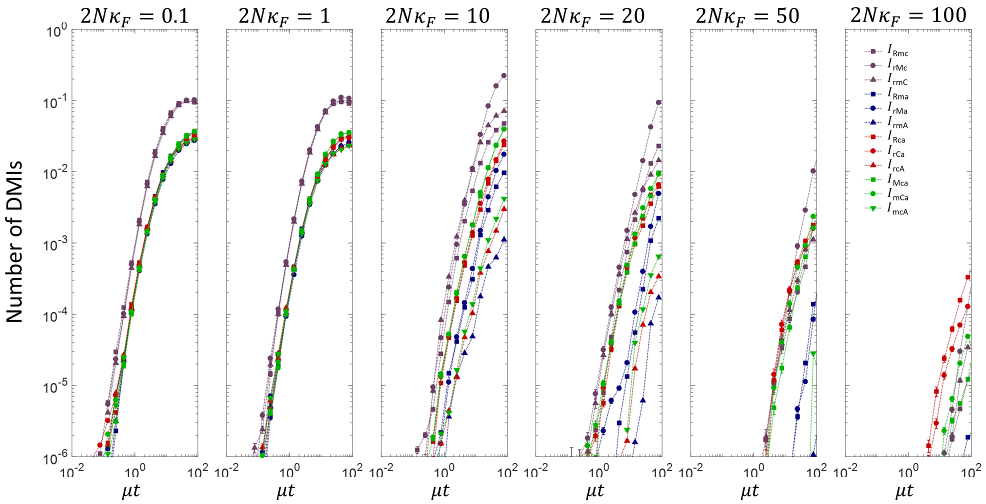

Fig S6. Plot of the spectrum 3-way DMIs vs divergence time for different scaled population sizes.

to the promotor to turn on transcription, through a high affinity interaction between the morphogen and the polymerase; the spatial position along the length of the embryo where the transcription switches from on to off is controlled by an interaction with the steepness of the morphogen gradient α. Given this, incompatibilities between R, M and C loci could arise through a 3-way interaction where the R locus interacts with the parts of the C and M loci coding for E_{RP} and $\tilde{E}_{RM}$, or where the M locus interacts with the parts of the C and R loci coding for E_{MB} and $\tilde{E}_{RM}$. So in analogy to 2-way DMIs, where a pair of loci give rise a single phenotypic binding energy trait, whose value contributes to fitness, here the triplet of loci, R, M and C, give rise to two binding energy traits, which together contribute to fitness. These two traits will co-evolve to maintain good fitness, balanced by the constraints of sequence entropy on the underlying 3 loci; at large population sizes, the effects of sequence entropy will diminish. On the other hand 3-way incompatibilities between, for example, M, C and α could arise due to an interaction of the E_{MB} binding energy trait with α; in this model this is subject to a sequence entropy constraint between only two loci. This is true for all the 3-way interactions that involve the α locus. Qualitatively, this then explains the behaviour at low population sizes, as sequence entropy dominates fitness, meaning that the behaviour of the different 3-way DMIs will be dominated by their underlying sequence entropy constraints.

The sequence entropy constraints for the 3-way interactions involving the α locus is straightforward and given by a binomial degeneracy function $\Omega(E) = \binom{\ell}{E/\epsilon}$, so that the sequence entropy function $S(E) = \ln(\Omega)$ is approximately quadratic in E, where here E represents one of the binding energies that interacts with α [S2]. However, for the other 3-way interactions that don't involve α, but involve the R, M and C loci, the sequence entropy constraint will be related to a degeneracy function $\Omega(E, \tilde{E}) = \Omega(E)\Omega(\tilde{E})$, where the joint number of sequences that give E and $\tilde{E}$ is a product, since these energy traits are coded by different sequences, even though they come from the same loci (the joint number of sequences $\Omega(E_{MB}, E_{MP}) \neq \Omega(E_{MB})\Omega(E_{MP})$ since the protein binding sequence of the morphogen that determines E_{MB} and E_{MP} is the same in this case). Given that the joint number of sequences that give E and $\tilde{E}$ is a product of two binomial coefficients, the sequence entropy function will approximately be a sum of two quadratic terms $S(E, \tilde{E}) \approx -\frac{2}{\ell_{pd}}(E/\epsilon_{pd} - \ell_{pd}/2)^2 - \frac{2}{\ell_{pp}}(\tilde{E}/\epsilon_{pp} - \ell_{pp}/2)^2$. At small population sizes, where genetic drift dominates selection, we expect the distribution of

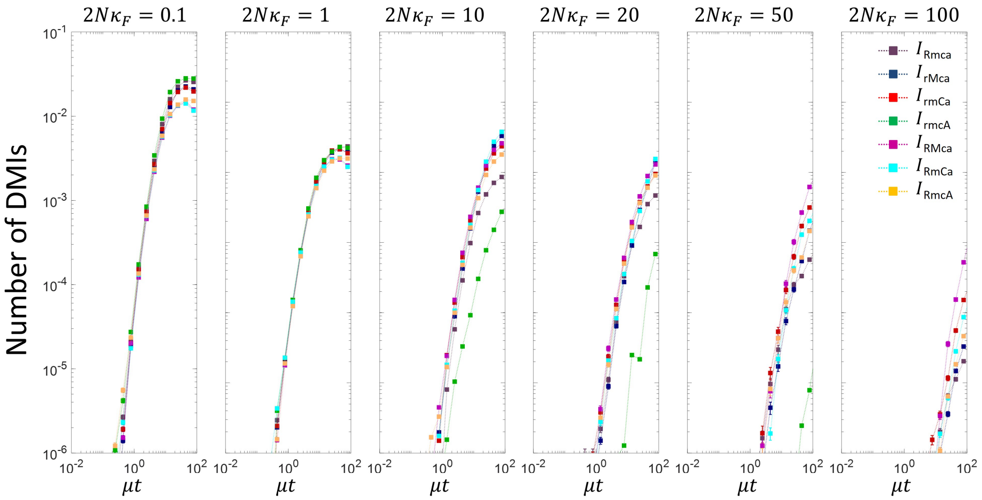

Fig S7. Plot of the spectrum 4-way DMIs vs divergence time for different scaled population sizes.

common ancestors to be such that they are poised at the incompatibility boundary for E and $\tilde{E}$; incompatibilities then arise when substitutions arise that take hybrids across the boundary.

Given that a 3-way DMI between the R, M and C genetic loci corresponds to co-evolution of a pair of binding energy traits, instead of a single binding energy trait for 2-way and 3-way DMIs that involve α, means the fraction of substitutions that lead to incompatibilities versus those that keep the hybrids compatible/fit becomes larger when going from one to two dimensions. This then explains why 3-way DMIs between the R, M and C loci gives rise to incompatibilities more quickly than those involving the α locus, as seen in Fig.S6.

4-way DMIs correspond to an interaction where all four loci require a particular combination of alleles for good patterning. As previously noted they are of much smaller number compared to 2- and 3-way DMIs, so here, we do not examine these DMIs in detail. However, we note that the 4-way DMIs shown in Fig.S7, show a similar pattern as found with 3-way DMIs, where for small scaled population sizes the DMIs tend to cluster, which suggests, as found for 2- and 3-way DMIs, this is due to sequence entropy constraints dominating the growth of DMIs; on the other hand, at large scaled population sizes this degeneracy is lifted and each hybrid has a different growth rate of DMIs, depending on their particular contribution to fitness and how that balances against the constraints of sequence entropy.

References

S1. Khatri BS, McLeish TCB, Sear RP. Statistical mechanics of convergent evolution in spatial patterning. Proc Natl Acad Sci U S A. 2009;106(24):9564–9569. doi:10.1073/pnas.0812260106.

S2. Khatri BS, Goldstein RA. A coarse-grained biophysical model of sequence evolution and the population size dependence of the speciation rate. Journal of theoretical biology. 2015;378:56–64.

S3. Khatri BS, Goldstein RA. Simple biophysical model predicts faster accumulation

of hybrid incompatibilities in small populations under stabilizing selection. Genetics. 2015;201(4):1525–1537.
